# Cardiac Involvement and Subsequent Death due to Extranodal NK/T Cell Cutaneous T-Cell Lymphoma: An Autopsy Case and Brief Review of the Literature

**DOI:** 10.30699/IJP.2021.139566.2524

**Published:** 2020-06-12

**Authors:** Nikolaos D. Goutas, Emmanouil I. Sakelliadis, Eleftheria Lakiotaki, Konstantinos D. Katsos, Kalliroi Spanou, Pinelopi Korkolopoulou, Dimitrios G. Vlachodimitropoulos

**Affiliations:** 1 *Department of Forensic Medicine and Toxicology, Medical School, National and Kapodistrian University of Athens, Athens, Greece*; 2 *Department of Pathology, Medical School, National and Kapodistrian University of Athens, Athens, Greece*; 3 Department of Pathology, 251 Airforce General Hospital, Athens, Greece

**Keywords:** Autopsy, Extranodal NK/T-cell lymphoma, Forensic medicine, Heart infiltration, Heart lymphoma, Nasal type, Sudden cardiac death

## Abstract

Cardiac tumors range from benign to high grade malignancies. The incidence of cardiac involvement either by primary, or secondary tumors during autopsy is reported to be extremely low. Extranodal NK/T-cell lymphoma (ENKTL), nasal type is an unusual type of lymphoma. The skin is the second most common site of involvement after the respiratory tract. We present a case of a 63-year-old male, who was recently diagnosed with ENKTL, nasal type, who received chemotherapy, and died without any evident cause. The corpse was referred for routine medicolegal examination. Macroscopical determination of the cause of death was not feasible and subsequent histopathological examination revealed heart infiltration by ENKTL that was found in vivo in cutaneous lesions. Similar infiltrations existed in the pancreatic tissue. To the best of our knowledge, myocardial infiltration of ENKTL, inducing severe myocardial lesions that eventually caused death, is rare, with limited cases reported in the literature.

## Introduction

Cutaneous lymphomas (CL) and specifically primary cutaneous lymphomas (PCL) represent a heterogeneous group of lymphoproliferative neoplasms, with either T/NK (CTNKCL) or of B (CBCL) cell origin (1).

PCL in general and especially CTNKCL, usually manifest in the skin without evident extracutaneous disease upon diagnosis (1). Classical CTCL (mycosis fungoides and variants, Sezary’s syndrome) and primary cutaneous CD30+ (anaplastic large cell lymphoma, lymphomatoid papulosis) include 90% of CTCL cases in the Western world. 

In the past, two distinct classification systems were employed by the World Health Organization (WHO) and the European Organization for Research and Treatment of Cancer (EORTC). Fortunately, a consensus was reached concerning a unique classification that would be able to resolve previous differences (2).

ENKTL is a predominantly extranodal lymphoma of NK-cell or T-cell lineage, characterized by vascular damage and destruction, prominent necrosis, cytotoxic phenotype, and association with Epstein-Barr virus (EBV). It is referred to as NK/T-cell lymphoma because although most cases appear to be genuine NK-cell neoplasms, some cases are of cytotoxic T-cell lineage (WHO). The disease is strongly associated with EBV, irrespective of the ethnic origin of the patients, indicative of the pathogenic role of the virus in ENKTL lymphomagenesis (3, 4).

## Case Presentation

In October 2015, a 63-year-old male was admitted to an Athens hospital, with cutaneous lesions suspected to be a lymphoma. He had history of diabetes mellitus, hypertension, dyslipidemia, atrial fibrillation, pacemaker and reduced renal function. He was diagnosed with cutaneous lymphoma, after a biopsy was performed. 

**Fig. 1:**
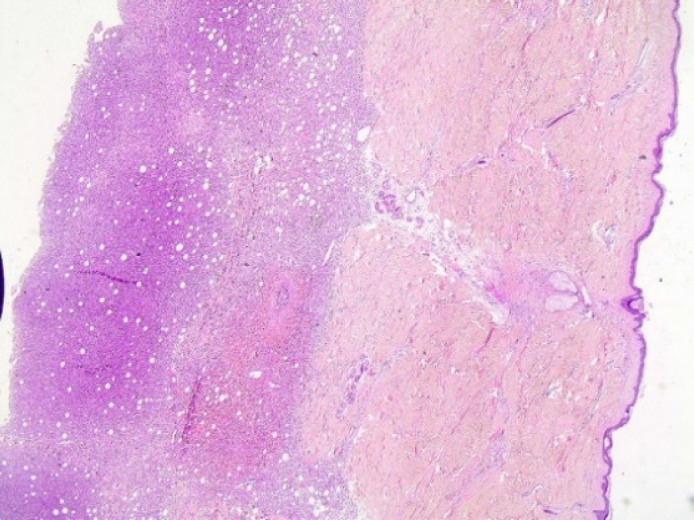
H&Ex20. In vivo skin lesion with dense infiltration of atypical lymphoid cells in subcutis

The in vivo pathology of the skin lesions (Figure 1) described that the subcutaneous tissue was densely infiltrated by an aggressive EBV+ cutaneous lymphoma, providing the differential diagnosis between ENKTL and primary cutaneous γδ T-cell lymphoma (PCGDTCL). No epidermal ulceration was noted. According to the pathology report, dermis was infiltrated as well. No significant vascular infiltration was observed (only small diameter vessels were affected). 

**Fig. 2:**
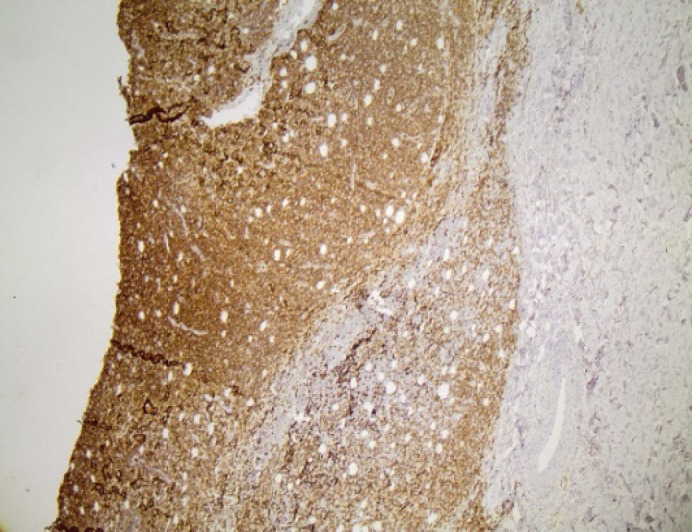
CD56x40. Diffuse intense cytoplasmic CD56 positivity was detected

**Fig. 3:**
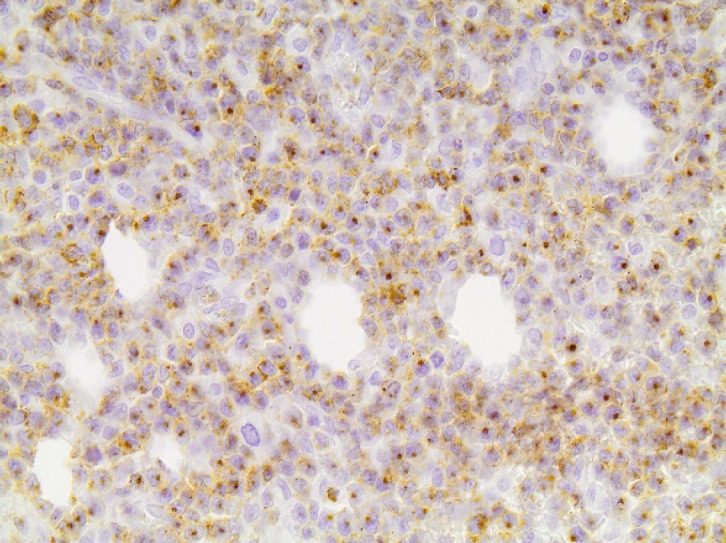
CD2x400. Diffuse intense cytoplasmic positivity was noted

**Fig 4:**
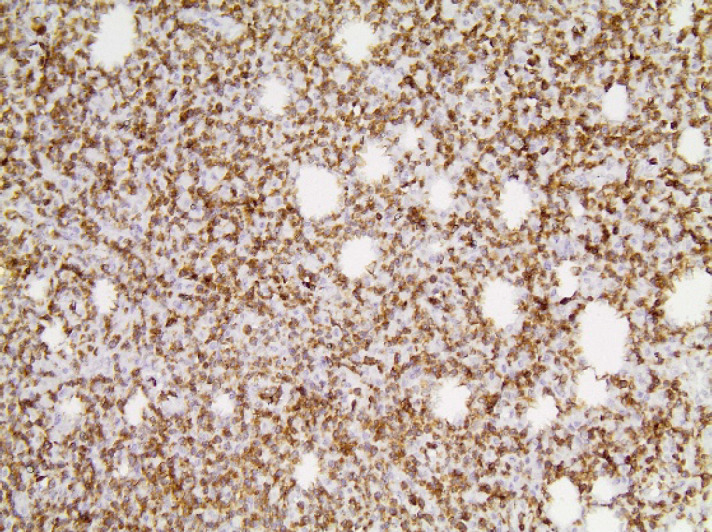
CD3x400. Diffuse intense cytoplasmic positivity was noted

**Fig 5:**
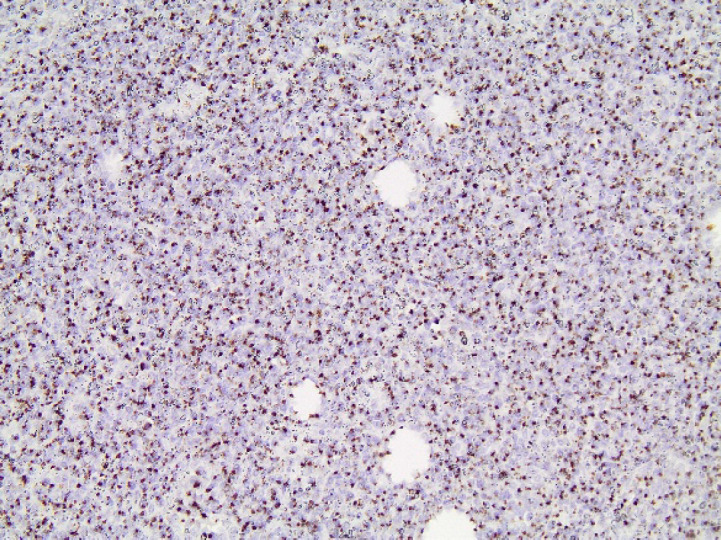
TIA-1x200. Diffuse granular cytoplasmic staining

**Fig. 6:**
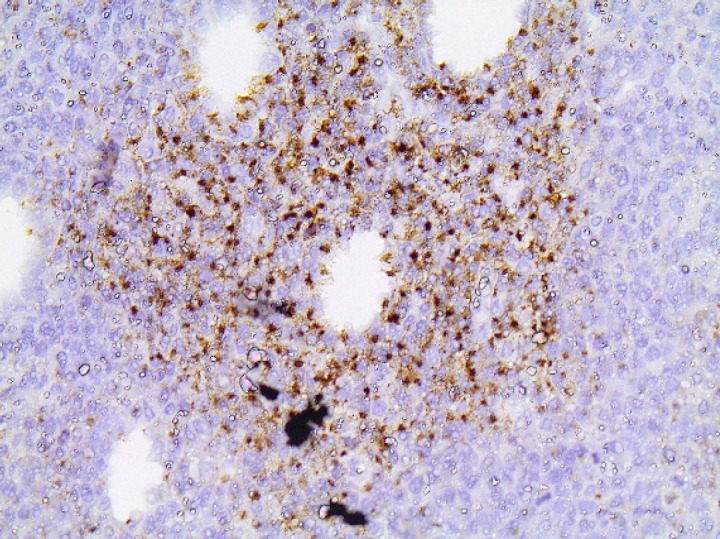
Granzyme Bx400. Diffuse granular cytoplasmic staining

**Fig. 7:**
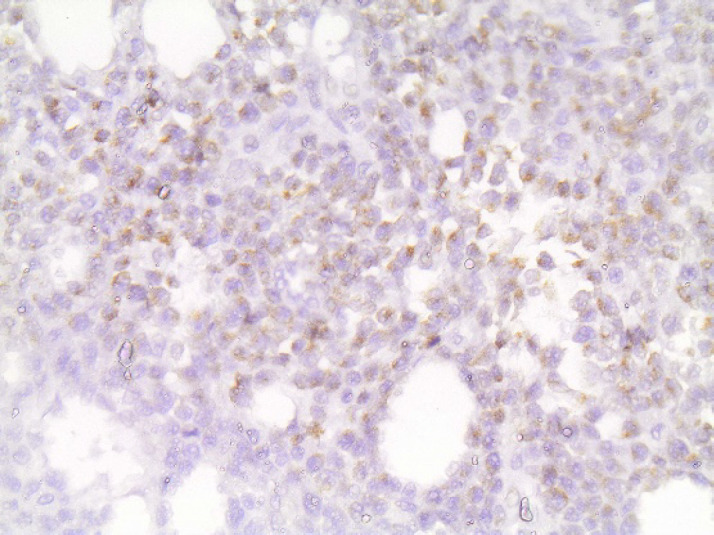
Perforinx400. Diffuse granular cytoplasmic staining

**Fig. 8:**
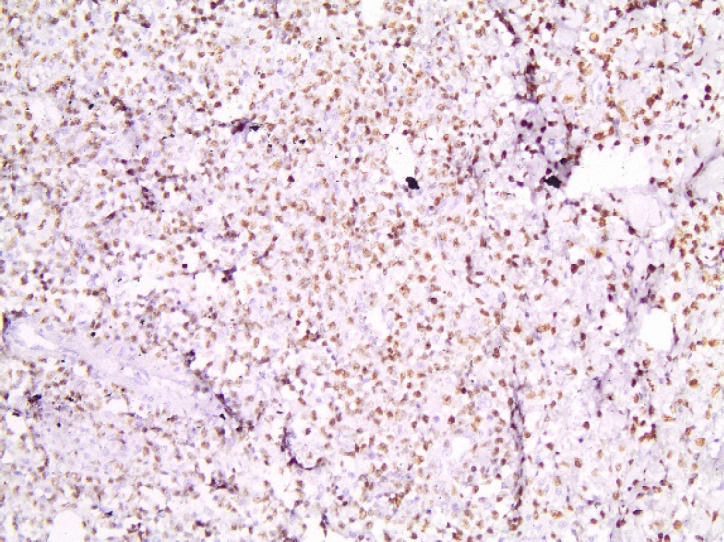
Ki67x100. Ki67 proliferation index was estimated at 80%.

**Fig. 9:**
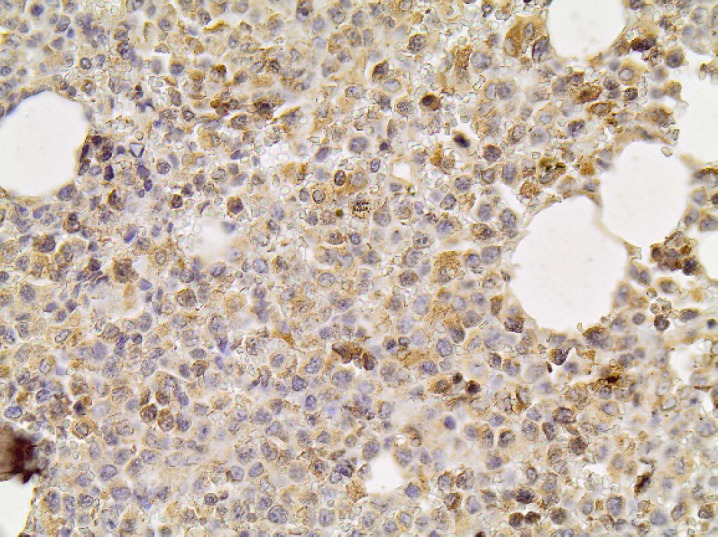
TCRγx400. Diffuse faint positivity to TRCγ immunostaining, while few cells are strongly positive

**Fig. 10:**
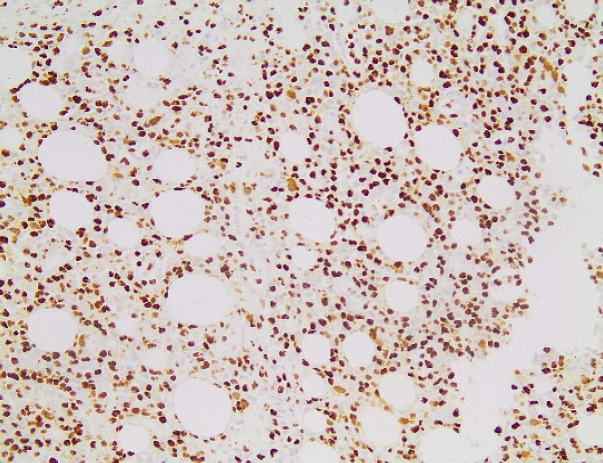
EBERx200. In situ hybridization for Epstein-Barr virus-encoded mRNA shows diffuse nuclear staining

The immunophenotype, which is summarized in Table 1, was as following: CD56+ intensively and diffusely (Figure 2), CD2+ (Figure 3) and CD3+ diffusely (Figure 4), CD4-, CD8-, CD5-, CD7-, TIA-1+ (Figure 5), Granzyme B+ (Figure 6), Perforin+ (Figure 7), βF1-, CD20-, CD79a-, CD138-, CD30-, κ-, λ-, MUM-1-. Ki67 proliferation index (Figure 8) was estimated at 80%. TCRγ expression was faint (Figure 9) and could not be considered diagnostic of a γδ-T cell lymphoma on its own. In situ hybridization for EBV-encoded mRNA (EBER) showed diffuse positivity of the neoplastic cells (Figure 10).

**Table 1:** Immunophenotype

CD56 (+) - intensively and diffusely
CD2 (+)
CD3 (+) - diffusely
CD4 (-)
CD8 (-)
CD5 (-)
CD7 (-)
TIA-1 (+)
Granzyme B (+)
Perforin (+)
βF1 (-)
CD20 (-)
CD79a (-)
CD138 (-)
CD30 (-)
κ (-)
λ (-)
MUM-1 (-)
Ki67 – 80%
TCRγ - faint
EBER - diffusely positive

For TR gene rearrangement analysis, tissue sections (5 μm) were deparaffinized with xylene and washed three times with 100% ethanol. After DNA extraction and quality assessment, multiplex PCR assay (TCR Gamma Rearrangement Ref: MAD-003994TP-2, Molecular Analysis Kit, Vitro Master Diagnostica, Vitro S.A. C/ Luis Fuentes Bejarano, 50, 41020-Sevilla, Spain) was applied using TCR primer sets targeting rearrangements of VJ loci of T cell receptor gamma chain and resulting in detection of peaks of significant height. Thus, the sample was proved to harbor TcRγ clonal rearrangements, indicative of a T-cell over NK- cell derivation.

According to the information provided by the clinicians, he was administered high doses of cortisone and diuretics. He was then administered cyclophosph-amide, anthracycline and etoposide. During the evening preceding death, the patient developed tachypnoea and acidosis, without previous evident underlying pulmonary pathology. Finally, the patient developed electro-mechanical dissociation and passed away. Clinicians suspected either sepsis or pulmonary embolism to be the cause of death.

**Fig. 11:**
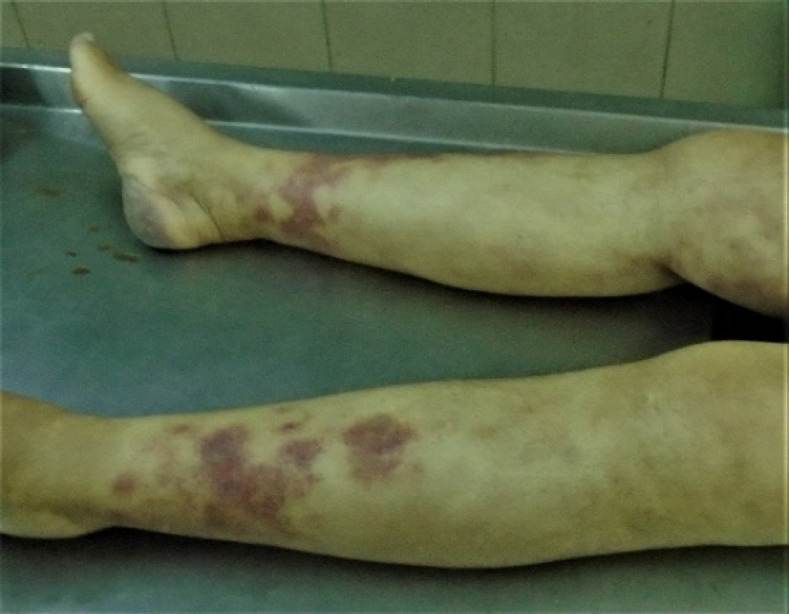
Macroscopic view of plaque-like skin on the extremities during the post-mortem examination

The corpse was submitted to routine medicolegal autopsy, which unfortunately did not lead to macro-scopical determination of the cause of death. Sepsis and pulmonary embolism, nevertheless, were already dismi-ssed from the morgue, as neither emboli, nor severe inflammation sites were detected, macroscopically. External examination revealed diffuse plaque-like skin lesions on the trunk and the extremities (Figure 11). Heart weight during autopsy was found to be 515 g. No neoplastic mass was detected macroscopically during the examination of all internal organs. The entire heart, along with tissue samples from the brain, lungs, liver, spleen, pancreas, and kidneys were collected for histopatho-logical examination.

**Fig. 12:**
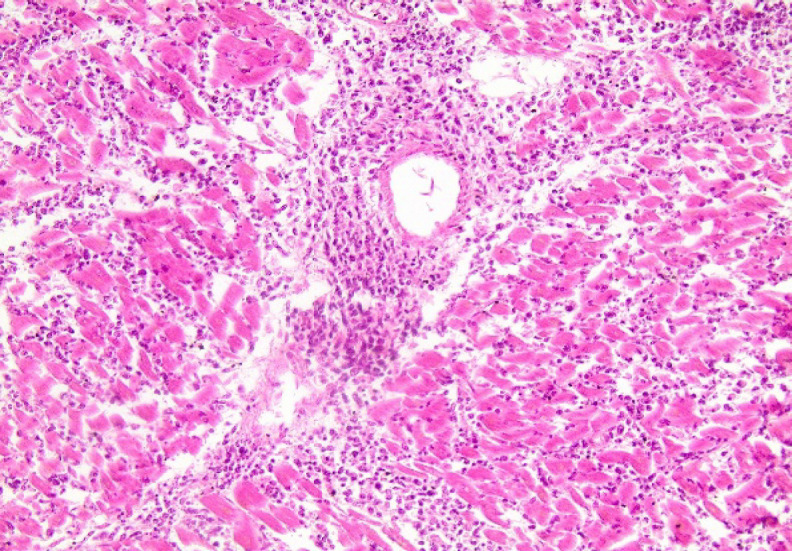
H&Ex200. Heart. The same neoplastic population detected in the skin, is destroying myocardial cells with prominent angiotropism

Microscopical examination revealed diffuse infiltration of the heart by atypical lymphoid population, with prominent angiocentricity and necrosis (Figure 12). The final depiction of angiocentric myocardial lesions was considered more suggestive of ENKTL over PCGDTCL. Areas of increased interstitial oedema were also detected, along with diffuse ischemic lesions of the myocardial tissue. The same atypical cells were detected in pericardial fat tissue as well. Furthermore, peri-pancreatic fat tissue was found to present the infiltration by the neoplastic population.

Myocardial cell necrosis due to lymphoma infiltration was the cause of death. It is very probable that myocardial infiltration led to arrythmias which caused the electro-mechanical dissociation reported by the clinicians.

## Discussion

ENKTL is highly aggressive, with short survival and poor response to therapy (5, 6). The upper aerodigestive tract (nasal cavity, nasopharynx, paranasal sinuses and palate) is the prototypical site of involvement and the other common sites include the skin, soft tissue, gastrointestinal tract and testes, while lymph nodes can be secondary infiltrated by lymphoma dissemination (7, 8). On the other hand, myocardium is rarely infiltrated with few cases described in the literature, causing heart failure and death in most cases (9, 10). 

In our case, the subcutaneous fat was densely infiltrated by atypical medium-sized lymphocytes with irregular nuclei and small prominent nucleoli that extended to the dermis, lacking epidermotropism. The neoplastic infiltrate showed areas of necrosis, but no angioinvasion was present. The neoplastic cells were diffusely positive for CD3, CD2, CD56 and cytotoxic molecules, double-negative for CD4 and CD8, while EBER was diffusely positive. TCRγ showed faint positivity in most neoplastic cells and was strongly positive only in a small number of neoplastic cells. 

ENKTL and PCGDTCL share many immune-morphological features and the differential diagnosis between them is often problematic. ENKTL is characterized by angioinvasion and angiodestruction, and EBV positivity and is regarded to be of NK lineage in most cases (11). As far as skin involvement is concerned, exceptions to the characteristic angioi-nvasive properties apply and non-ulcerated lesions, such as the one we are describing, can show minimal or absent angiocentricity. (12) Furthermore, although most cases are of NK lineage, rare γδ-Τ-ENKTL cases are also described in the literature, presenting TCR clonality in molecular studies (6).

PCGDTCL cases also often feature angiodes-truction and necrosis and are always characterized by strong TCRγ positivity and βF1 negativity. This lymphoma type is considered EBER-negative, but there are reports in the literature that have reported EBER-positive extranodal γδ Τ-cell lymphomas in sites other than the skin (11).

In our case, the diffuse positivity for EBER was more suggestive of ENKTL over PCGDTCL. However, the angiocentricity was lacking from the initial skin biopsy and was depicted in sections from the myocardium. TCRγ faint positivity was finally not regarded as a strong clue in the diagnosis of PCGDTCL, as it does not exclude ENKTL of γδ Τ-cell origin.

Diagnosed cardiac tumors range from benign to high grade malignancies. A classification in three groups is employed: pediatric tumors (mainly hamar-tomas), benign tumors and malignancies (primary cardiac sarcomas, lymphoma and metastases) (13). Incidence of cardiac involvement by primary or secondary tumors during autopsy is reported to be very low, more specifically 0.056% and 1.23%, respe-ctively. 

Primary tumors in adults may arise more frequently from the endocardium, while in metastatic tumors, pericardium is the most frequent site of origin (for epithelial malignancies), with endocardium being limi-ted only to some (tumors growing in great veins) (13).

Cardiac lymphomas, mainly in the context of disseminated disease, may be present in up to 10-20% of patients diagnosed with non-Hodgkin lymphoma (NHL) (13). 

Primary cardiac lymphoma represents 1.3% of all primary cardiac tumors and approximately 0.5% of extranodal lymphomas. Lymphomas manifest with atrial infiltration, followed by infiltration of the ventricular walls (13). 

As explained above, the patient died of myocardial necrosis due to lymphoma infiltration of the heart. To the best of our knowledge, there are rare reports so far in available literature, describing infiltration of the heart by ENKTL (14, 15).

## Conclusion

It is essential for clinicians to take under consideration that ENKTL may involve the heart, as it may lead to sudden cardiac death due to disruption of the cardiac conduction system. Our case report, which presents direct heart and pancreatic involvement by ENKTL should lead to improved cardiac monitoring of patients, to prevent fatal cardiac events.

In general, visceral infiltrations are obviously critical for overall survival. Moreover, heart involvement by ENKTL may prove to be directly lethal and may explain previously unexplainable sudden death in relevant cases.
